# Form informs function: innervation patterns of projection neurons correspond to response profiles of mushroom body output neurons during olfactory-visual integration

**DOI:** 10.3389/fphys.2026.1898182

**Published:** 2026-08-18

**Authors:** Athil Althaf Aliyam Veetil Zynudheen, Andrea Rafaela Nicolaidou, Claudia Groh, Volker Dürr, Wolfgang Rössler, Martin Strube-Bloss

**Affiliations:** 1 Biological Cybernetics, Department of Biology, Bielefeld University, Bielefeld, Germany; 2 Behavioral Physiology and Sociobiology (Zoology II), Biocenter, University of Würzburg, Würzburg, Germany

**Keywords:** bimodal integration, honeybee, mushroom body output neurons, olfaction, vision, feed-forward inhibition, feed-back inhibition

## Abstract

Several studies have shown that honeybees have a remarkable ability to detect, learn, and discriminate different floral qualities such as colour patterns and odour bouquets. However, how they integrate this multi-sensory information at the neuronal level is less understood. The sites for multi-sensory convergence in honeybees and other insects are the mushroom bodies (MB). In this study, we focus on MB output neurons (MBON) to examine how different MBONs process olfactory, visual, and olfactory-visual compound stimuli using extracellular multi-unit recordings. By analysing functional responses, we defined three MBON subpopulations: “bimodal”, “unimodal light”, and “unimodal odour”. All three groups exhibit compound-mediated modulation in both directions, meaning that compared to the unimodal response, the response strength to the compound can be either higher or lower. This suggests the presence of a separate compound-activated pathway, especially for unimodal MBONs. Comparing our results to the morphology of the MB input regions, the calyces, which are both divided into the lip (olfaction), collar (vision), and the basal ring (bimodal), we provide evidence that the latter region might play a key role in compound signalling. By establishing a model based on information flow through the MB circuitry, we reproduced the functional phenotypes exhibited by the different MBON types.

## 1 Introduction

Plant-pollinator interactions are a fascinating puzzle of co-evolution, as the survival of both parties is intertwined through pollination. While plants have evolved reproductive organs like flowers that provide various sensory cues (e.g. olfactory, visual, or tactile), pollinators have developed the sensory faculties to detect and pursue these cues for foraging pollen or nectar. Different sensory cues have differing spatio-temporal properties. Although gustatory and textural cues are contact-based, visual and olfactory cues provide *near-range* guidance and *far-range* detection (Rands et al., 2023). Visual cues of a flower are usually saturated bright colours, which provide a spectral contrast against the background, and olfactory cues are mainly volatile organic compounds, which are carried with the air as odour filaments that are detected by antennal olfactory receptors. Although generalist pollinators, honeybees associate food quality and quantity with a visited flower, establishing flower constancy (Waser, 1986), a phenomenon where pollinators revisit the reward-associated flowers even when other reward-containing alternatives are present. While flower constancy can be advantageous in increasing foraging efficiency, it remains largely unclear which aspects of floral perception enable this. One strategy is to integrate different sensory information, such as the floral colours and scents, which effectively makes these particular flowers “stand out” from the other alternatives.

Conditioning experiments have shown that bees are capable of learning olfactory-visual compound stimuli and differentiating them from the unimodal elements (Mansur et al., 2018; Strube-Bloss et al., 2023). Furthermore, bimodal cues significantly enhance learning performance when the unimodal stimuli are of weaker intensities (Gil-Guevara and Riveros, 2024). In the honeybee brain, the regions associated with multisensory integration, learning, and memory formation are the mushroom bodies (MB). They primarily receive prominent input from both the olfactory and visual projection neurons (oPN and vPN, respectively) innervating the MB calyx at distinct input regions: the lip, collar, and basal ring. The lip receives input from oPNs (Kirschner et al., 2006; Groh and Rössler, 2020; Habenstein et al., 2023), while the collar receives input from vPNs. In addition, the basal ring receives input from both oPNs and vPNs, underscoring its potential to contribute to olfactory-visual integration (Gronenberg, 2001; Groh and Rössler, 2020). At the input side, these few hundred oPNs and vPNs are connected to approximately 184,000 Kenyon Cells (KC), the intrinsic neurons of the MB, in each brain hemisphere (Kenyon, 1896; Witthöft, 1967). KCs can be subdivided into “class I inner compact, spiny KCs” innervating the basal ring (olfaction and vision), “class I inner non-compact, spiny KCs” innervating the lip (olfaction) and collar (vision), and an anatomically distinct “class II outer, clawed KCs” with claw-like dendritic specialisations innervating all three input regions of the MB calyx (Strausfeld, 2002; Nicolaidou et al., 2026). The concentric organisation of KC dendrites in the calyx is maintained within distinct layers of the vertical lobe (VL) of the MB formed by KC axonal terminals. From these distinct KC layers, about 400 MBONs receive convergent information from the ~184,000 KCs (Rybak and Menzel, 1993; Strausfeld, 2002). MBONs can be subdivided into 7 morphological subgroups (Rybak and Menzel, 1993). Among these, A6 and A7 are bilateral neurons, i.e., their projections extend to both brain hemispheres, suggesting involvement in integrating information across both sides. The rest of the neurons (A1-A5) are unilateral, i.e. arborise in a single brain hemisphere. Within this group of neurons, A3 (110 in total) are γ-aminobutyric acid (GABA)-ergic neurons that form inhibitory recurrent connections within the MB (Rybak and Menzel, 1993). Among A3, there are two different clusters, the first comprises feedback neurons that receive input from the VL and inhibit the MB calyx (A3v, part of the “ventral” soma cluster), and the second are the lobe connecting neurons that connect locally within VL and the medial lobe (A3d, part of the “dorsal” soma cluster).

When characterising subpopulations of MBON response in our earlier studies, we were able to show that single MBONs were recruited to encode odour-reward associations (Strube-Bloss et al., 2011), predict behavioural performance of single animals (Strube-Bloss et al., 2021), and integrate learned spatial information (Strube-Bloss et al., 2016). Furthermore, at this processing level, MBONs integrate information from different sensory modalities, such as olfaction and vision, to categorise incoming information (Strube-Bloss and Rössler, 2018). The latter is accomplished by different subpopulations of MBONs: Some are sensitive to a single modality (olfaction or vision), whereas others are bimodal, responding to both modalities. The latter seemed to show additive, but non-linear computation of the presented compound stimuli, but were not analysed further. Therefore, the objective of our present study is to further characterise these bimodal and unimodal MBONs and to link their response profiles to the overall MB circuitry. We do so by means of extracellular multi-unit recordings in alive and behaving honeybees, which we stimulate with various visual, olfactory and olfactory-visual compound stimuli. By examining the rate responses and latencies of MBONs to two compound stimuli and their unimodal elements, respectively, we further categorised MBONs into unimodal light, unimodal odour, and bimodal MBONs. All three groups showed a nonlinear up- and down-modulation during compound stimulation. In particular, the down-modulation suggests that the underlying connections include the aforementioned inhibitory connections of the A3 MBON cluster. Incorporating these findings and other morphological aspects, along with knowledge of information flow from the PN level through the olfactory and visual pathways to the MB, we established a rate-based model that reproduces the measured MBON response profiles.

## 2 Methods

### 2.1 Animal handling and preparation

Pollen-carrying honeybee foragers were caught at the hive entrance, immobilised on ice, and restrained in a cylindrical metal holder in a way that they were free to move their antennae and extend their proboscis (Bitterman et al., 1983). They were fed one drop of 50% sucrose solution (volume by volume) and kept in a humid container overnight. The next morning, only motivated bees, which showed a lively proboscis extension, were chosen for the experiments. Their heads were fixed with dental wax beneath the eyes. Low melting-point wax (Eicosane) was added to fix the scapi of both antennae, leaving the flagellum free, but preventing bigger antennal movements. A small rectangular window was cut between the midline of the central ocellus and halfway to the scape, just up to the rim of the compound eyes. Head glands, fat body and tracheae were removed to expose the brain, allowing a clear identification of the VL (Rössler and Brill, 2013).

**Figure 1:**
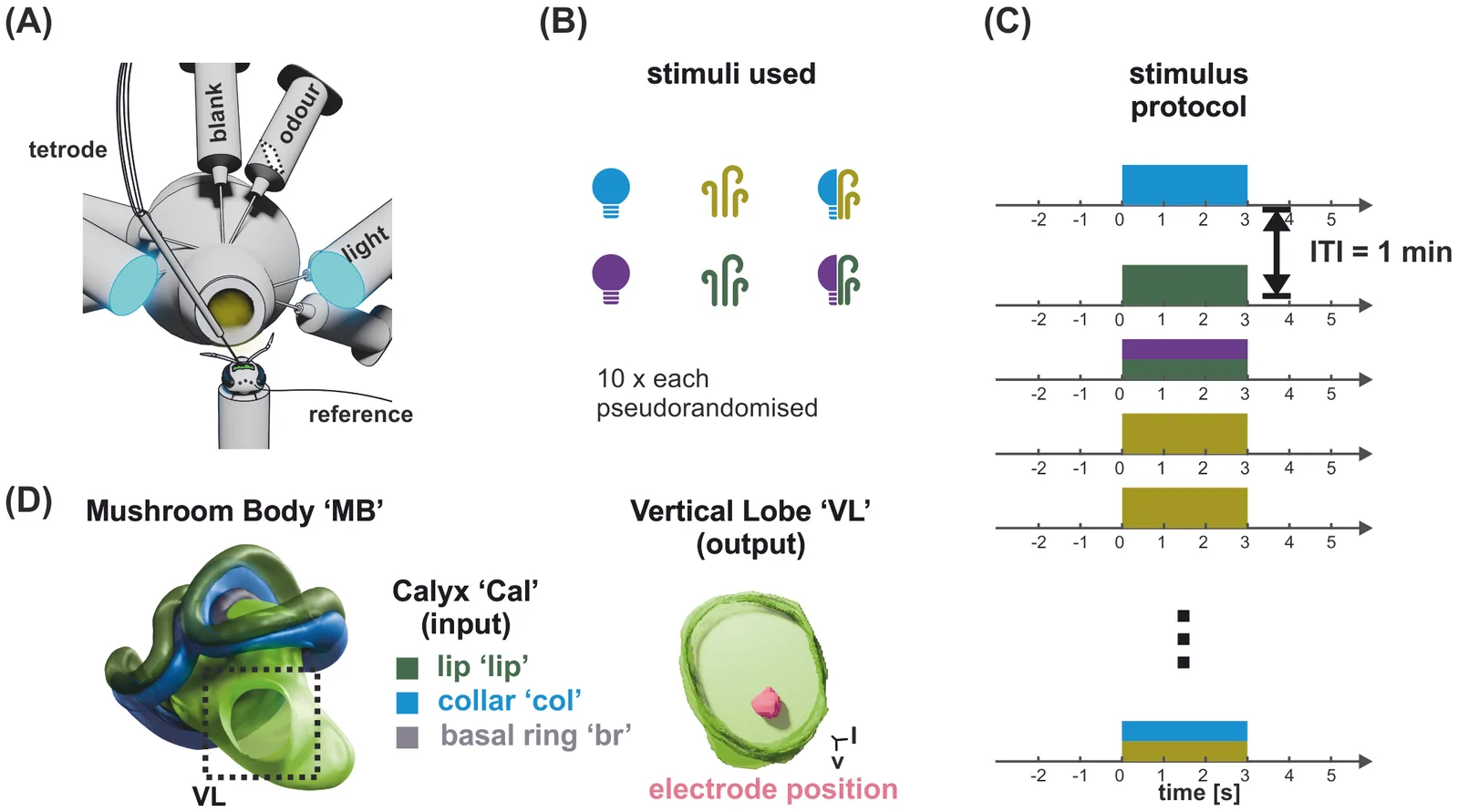
Experimental setup, design and electrode reconstruction: **(A)** Experimental setup with an olfactometer providing different odours and monochromatic light stimuli. The tetrode is inserted into the ventral part of the vertical lobe (VL) of the honeybee brain. **(B)** Two wavelengths (blue and UV), two odours (peppermint, farnesol) and their odour-light compounds (blue + peppermint, UV + farnesol) were presented. **(C)** The stimulus presentation lasted 3 (s) In addition, we recorded 3 s of pre- and post- stimulus time, respectively. Each stimulus was repeated 10 times in a pseudorandomised sequence with an inter-trial interval (ITI) of 1 min. **(D)** *Left:* The mushroom body (MB) of a honeybee (adapted from Habenstein et al., 2023, licensed under CC BY 4.0.), including its input region, the calyx, divided into the lip (lip), the collar (col), and the basal ring (br), and its output region, VL, dashed square. *Right*: Example 3D reconstruction of the electrode position within the VL (l = lateral, v=ventral).

After fixation, a tetrode was gently inserted into the ventral VL (**Figure 1D**) using a micromanipulator, to record signals at a depth of 100–200 µm. Once clear spiking signals appeared, the head capsule was sealed with two-component silicone (KWIK-SIL, World Precision Instruments, Florida, USA) to prevent brain from dehydration and to fix the electrode in place.

### 2.2 Olfactory stimulus

Olfactory stimuli were presented using a custom-made olfactometer, as used in previous studies (Schmalz et al., 2022; Strube-Bloss and Rössler, 2018). Two syringes were inserted pairwise into a permanent airflow (1.5 m/s), one of which was equipped with an odour-soaked filter paper, while the other one was left blank (**Figure 1A**). During odour stimulation, an optocoupler-driven solenoid valve flipped the airflow from the blank to the odour-containing syringe. The odours used were peppermint and farnesol (Sigma-Aldrich Chemie GmbH), diluted (1:100; volume by volume) in paraffin oil (Sigma-Aldrich Chemie GmbH). The chosen odours were selected for their presence in floral scents and pheromones and are also used in olfactory conditioning studies (Okada et al., 2007; Williams et al., 1981). Five microliters of the diluted odour were infused on the filter paper (2 x 2 cm) and placed inside the syringe. The outlet of the olfactometer was placed ~2cm away from the bee, facing the antenna (**Figure 1A**). An exhaust hood was placed behind the bee to prevent odour accumulation in the recording arena.

### 2.3 Visual stimulus

Two monochromatic LED lights (blue: 435 nm and UV: 355 nm) were placed at the end of a transparent plexiglass rod (diameter: 10 mm, length: 100 mm) to diffuse the light. Their power output at the front of the rod was adjusted to 15 µW/mm^2^ using a laser power meter. Two such rods were placed 2–3 cm away from the bee, each facing one of the compound eyes, respectively. The wavelengths were chosen to be consistent with the prior studies investigating the visual response of MBONs (Schmalz et al., 2022; Strube-Bloss and Rössler, 2018) (**Figure 1A**).

### 2.4 Stimulus protocol

During the experiments, we presented 2 different lights, 2 different odours, and 2 odour-light compounds (blue + peppermint, UV + farnesol) (**Figure 1B**). Each stimulus was presented 10 times, shuffled in a pseudo-randomised manner so that no stimulus of the same kind was repeated more than twice in a row. A single trial was recorded for 9 seconds, consisting of 3 seconds pre-stimulus, 3 seconds of stimulus, and 3 seconds of post-stimulus time. Each trial was separated by a 1-minute inter-trial interval, and the entire experiment lasted approximately 70 minutes (**Figure 1C**). The trial sequence was generated by a custom MATLAB script, which created configuration files for the Neuralynx trial control software that synchronised data acquisition with stimulus presentation via TTL pulses. The compound stimulus was synchronised using an Arduino that turns on both the single light and odour channels simultaneously when receiving a TTL signal from Neuralynx trial control.

### 2.5 Electrophysiology and data acquisition

We adapted our standardised extracellular recording protocol (Brill et al., 2014; Strube-Bloss and Rössler, 2018; Schmalz et al., 2022). Tetrodes were custom-made by glueing together four insulated copper microwires (15µm diameter, polyurethane-coated). A silver wire (25µm in diameter) served as a reference and was inserted between a compound eye and the outermost ocellus. First, each tetrode channel was recorded with reference to the silver wire (T1-T4). In a second step, we differentiated between the pairwise combination of T1-T4 to ensure that we record only neurons close to the electrode tip. The best four combinations of the differentiated signals showing pronounced action potential waveforms were recorded at a sampling rate of 30 kHz and pre-filtered with a bandpass filter (250–9000 Hz), using Cheetah Data Acquisition software (Neuralynx, Bozeman, Montana, USA).

### 2.6 Spike sorting

The raw voltage traces acquired were exported into Spike2 (Cambridge Electronic Design, Cambridge, UK). A semi-automatic spike-sorting workflow as described earlier (Strube-Bloss et al., 2011, 2012, 2016; Brill et al., 2014) was implemented to extract single-unit activity. In short, spike events were detected by setting a manual threshold on all four differentially recorded voltage traces, using the tetrode sorting function implemented in Spike2. Waveforms of threshold-crossing events were used to create templates across all 4 tetrode channels. Based on template matching, individual spike events were assigned to these individual templates. To visually inspect unit separation, we performed a principal component analysis (PCA), where individual waveform combinations across tetrode channels formed clusters in the space spanned by the first 3 PC axes. The individual waveforms assigned to each cluster were assumed to be produced by an individual neuron and therefore called a ‘unit’. The timestamps of all spikes obtained from an individual unit were further analysed in MATLAB (version R2022b, MathWorks). In total, we extracted 140 units from 43 animal preparations.

### 2.7 Visualising recording position

Before being inserted into the ventral VL, tetrodes were dipped into the fluorescent stain Alexa Fluor 647 Hydrazide (A20502, Thermo Fisher Scientific GmbH, Dreieich, Germany) for 20 minutes. After a successful recording, tetrodes were slowly removed, the bee brain was carefully excised from the head capsule and fixed in Fix-Mix (2% formaldehyde/2% glutaraldehyde) in phosphate-buffer saline (PBS) for 3 days. After that, the brain was washed again in PBS (5 times with 10-minute intervals), followed by serial dehydration in ethanol (30%, 50%, 90%, 95%, 100%). Later, the brain was cleared in methyl salicylate and mounted on a slide. We used a confocal microscope (Leica TCS SP8 MP, Leica Microsystems AG, Wetzlar, Germany) in 647nm (for electrode position) and 555nm (for autofluorescence) at 43x magnification to image the brain samples (Supplemental **Supplementary Figure 1**).

### 2.8 Electrode reconstruction

The confocal images were saved as a hyperstack, with channels containing Alexa 647 Hydrazide (for the electrode staining) and autofluorescence (for the brain). After importing the image into FIJI (Schindelin et al., 2012). The contrast of each channel was manually adjusted, and the channels were merged. This stack was imported into a blank TrakEM2 (Cardona et al., 2012) template, and two area lists were created. The area of the electrode staining was traced frame by frame (skipping 2–3 frames). The “interpolate all the gaps” function was used to fill in the in-between frames, and the results were later inspected and edited to correct interpolation errors. The same procedure was repeated for tracing the VL region. Both area lists were exported in 3D format and later visualised in Blender, an open-source 3D software (https://www.blender.org/) (**Figure 1D**).

### 2.9 Statistics and data analysis

#### 2.9.1 Calculating mean firing rate

**Figure 2:**
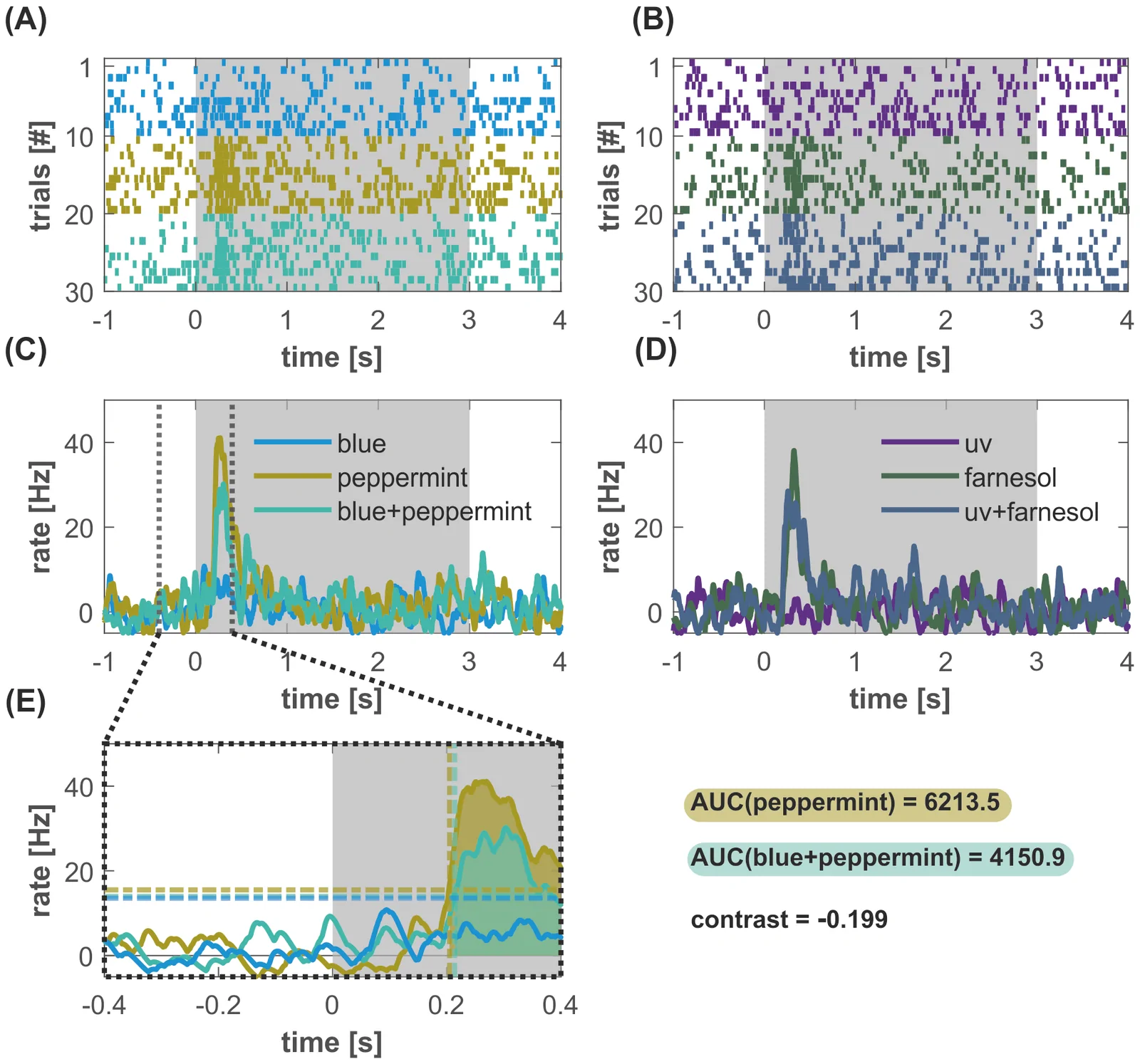
Example MBON activity and contrast calculation: **(A, B)** Colour-coded spiking activity of an individual unit in response to different lights, odours and compound stimuli [for colour codes see legends in **(C, D)**]. Grey shading marks the stimulation time. **(C, D)** Baseline corrected averaged firing rates across the 10 repetitions of each stimulus revealed that this example unit did not respond to the light stimuli but showed an increasing firing rate when stimulated with the odours. Both odour-light combinations evoked decreased activity compared to the individual odour response. **(E)** Rate response during the response detection window, 0.4 s after stim on (grey), zoomed in from **(D)** Coloured horizontal dashed lines marking the individual response detection threshold (5xσ). The timestamps (vertical dashed lines) of crossing events were used for the latency analysis. As a measure of response strength, we calculated the stimulus-dependent integral of the mean response rate of each individual unit within the first 0.4 seconds starting with the threshold crossing, expressed as area under the curve (AUC; cp. coloured shades/numbers).

Timestamps of each stimulus onset were aligned with the spike train (timestamps of an action potential of the individual unit) to separate individual trials, such that each trial included 1 second of pre-stimulus time, 3 seconds of stimulus and 1 second of post-stimulus time (**Figures 2A, B**). Each individual trial was then convolved with an asymmetric alpha kernel to obtain the instantaneous firing rate of the unit at this trial (Nawrot et al., 1999). The 10 trials of each stimulus repetition were averaged and baseline corrected by subtracting the mean firing rate of the pre-stimulus time from the whole firing rate trace (**Figures 2C, D**).

#### 2.9.2 Response detection and categorisation of MBON behaviours

**Figure 3:**
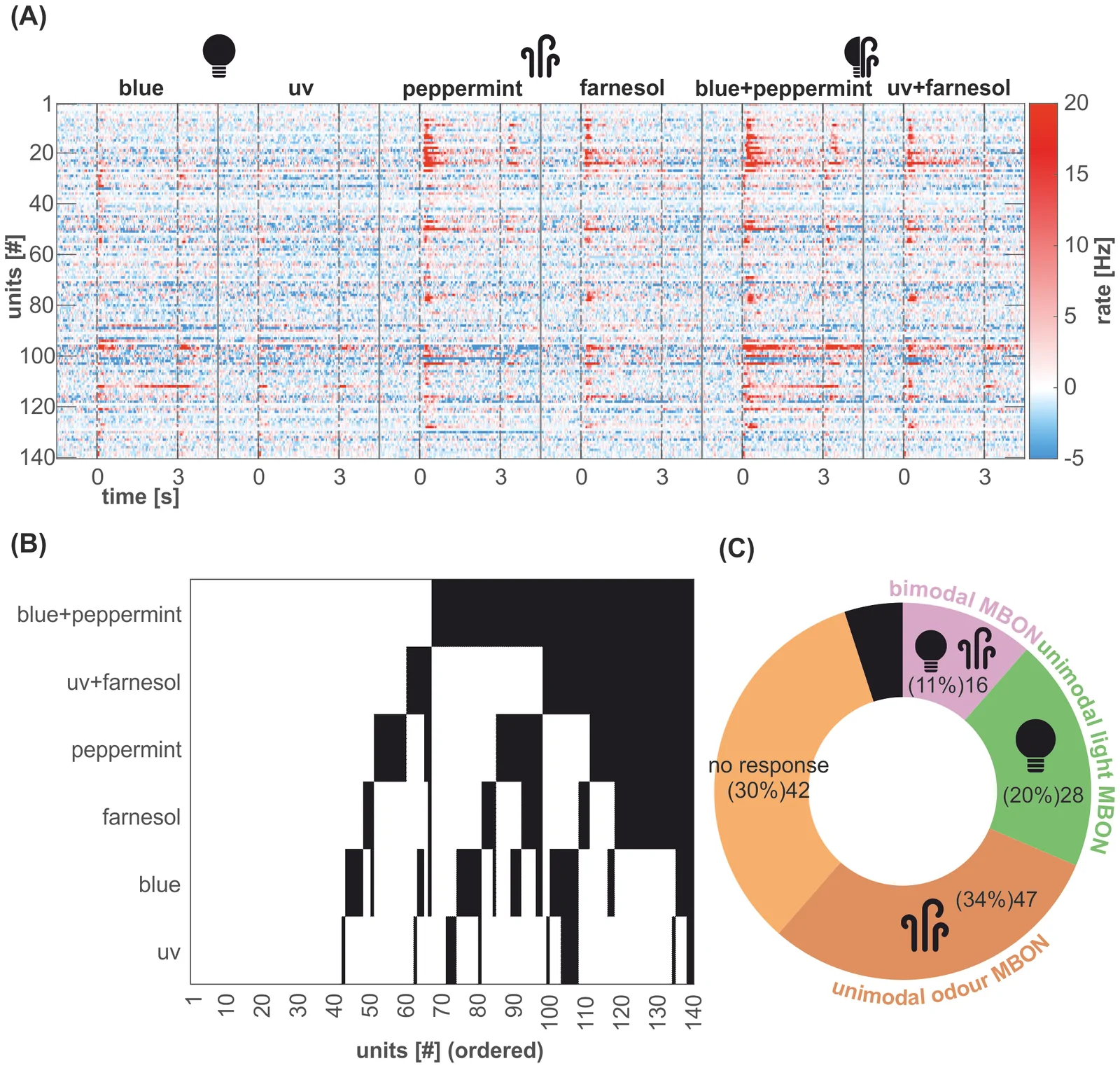
Population response pattern and functional categorisation: **(A)** Heatmap of the population responses of 140 potential MBONs out of 43 bees. Each row corresponds to an average instantaneous firing rate of a single unit in response to visual (left), olfactory (middle), and olfactory-visual compound stimuli (right). Vertical lines separate stimuli and dashed lines mark stimulus on- (0 s) and off-sets (3 s). **(B)** Stimuli-dependent binary response matrix of all 140 units (response=black; no response=white). Units were ordered based on the responses to the compound stimuli, starting with responses to both compounds from the right. **(C)** Proportion of the different response types. About 20% of the units responded to one or both wavelengths of light (unimodal light MBONs), 34% responded to one or both odours (unimodal odour MBONs), and 11% were sensitive to at least one odour and one light stimulus (bimodal MBONs). In addition, about 30% did not respond to any of the stimuli. However, 7 MBONs (black section) responded only when the two modalities occurred together.

A unit’s response was defined as such if its mean firing rate after stimulus onset (time window 0 to 0.4s) was higher than 5 times the standard deviation (σ) of the basal firing rate before stimulus onset (time window -0.4s to 0s; see **Figure 2E**). The cutoff (5xσ) and the time window (0-0.4 s) were chosen to be sensitive enough to resemble the ground truth while avoiding any noisy threshold-crossing events from being registered as a response. If a unit responded to either one of the two light sources, but not to an odour, it was categorised as a “unimodal light” MBON. Similarly, a unit responding to either of the odours, but not to light, was categorised as a “unimodal odour” MBON. Units that were responsive to both light and odour stimuli were categorised as “bimodal” MBONs. Units that never crossed the response threshold were categorised as “non-responsive” (**Figure 3C**).

#### 2.9.3 Latency detection

Furthermore, we extracted the timing of the threshold crossing of the individual units and compared the modality-dependent response latencies of the different MBON categories, using a Friedman test for matched samples. In bimodal MBONs, the Tukey-Kramer test was used as a *post-hoc* test to assess significant differences between the groups.

#### 2.9.4 Calculating bimodal and unimodal contrasts

To analyse how bimodal MBONs integrate the visual and olfactory pathways, we compared the response strength induced by the single modalities to the compound-induced response strength by measuring contrast for additivity. This is a measure to determine how different the response strength for the compound was with respect to the sum of the response strengths of the individual modalities. Since there were two sets of compound stimuli and the constituent unimodal stimuli, we calculated the contrast measures separately for each set. As a measure of response strength, we calculated the stimulus-dependent integral of the mean response rate of each individual unit during the first 0.4 seconds after stimulus onset, expressed as the area under the curve (AUC, **Figure 2E**). We used only bimodal MBONs in which we could detect a response to the compound (N = 15), excluding bimodal MBONs not passing our response criteria during compound stimulation (N = 3). The equation used to calculate the contrast for additivity is as follows:


$$Contrast=\frac{A_{C}-\left(A_{L}+A_{O}\right)}{A_{C}+\left(A_{L}+A_{O}\right)}$$

**Figure 4:**
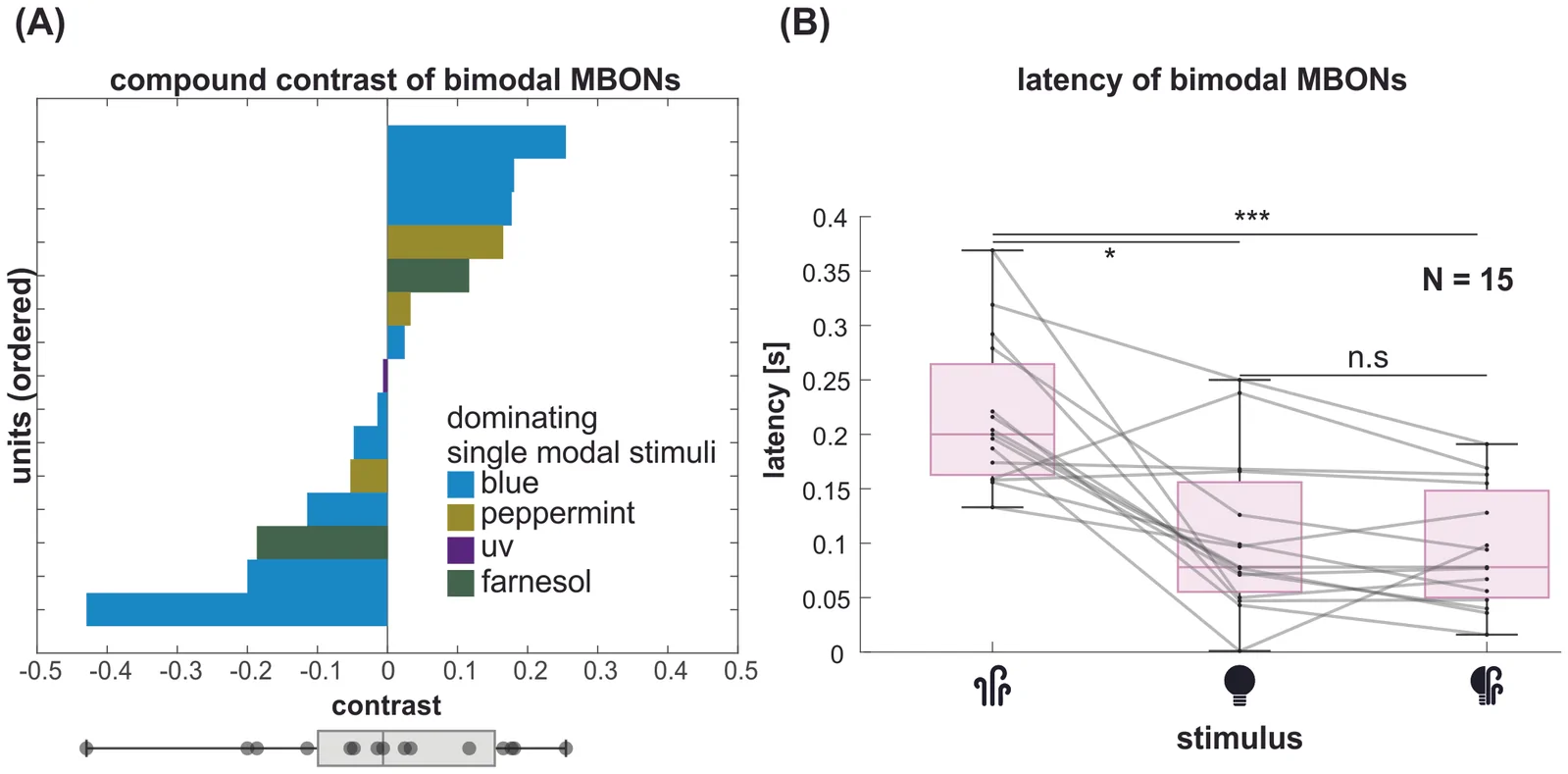
Compound-mediated modulation in bimodal MBONs: **(A)** Compound contrasts of the bimodal MBONs. Each bar is the contrast of a single unit representing the relative response difference between the compound and the sum of the single modalities. Colours indicate the single modality that had the highest response. All contrast values are shown in the boxplot below to illustrate variation among different bimodal MBON units. **(B)** Their stimulus-dependent latency distributions showed that the pure odours induced significantly slower responses compared to pure light or odour-light compounds (Friedmann test, p<0.001***, *post-hoc* Tukey-Kramer test: p<0.05*; p<0.001***), whereas pure light stimuli and odour-light compounds evoked the same latencies (*post-hoc* Tukey-Kramer test: n.s.). Lines connect the latencies of individual units.

Where *A* is the AUC of the positive rate response to the compound (C), light (L), and odour (O), respectively. The closer the contrast is to 0, the more likely the MBON is to add up the response strengths of the individual modalities (see **Figure 4A**).

Since we observed that MBONs categorised as unimodal also showed an increased or decreased response strength when stimulated with a compound (unimodal light and compound N = 18; unimodal odour and compound N = 39), but we could not add the sum of both modalities, we used a different contrast measure to assess how similar the unimodal response is to the compound response, calculating the contrast as follows:


$$Contrast=\frac{A_{C}-A_{L}}{A_{C}+A_{L}}$$

or


$$Contrast=\frac{A_{C}-A_{O}}{A_{C}+A_{O}}$$

**Figure 5:**
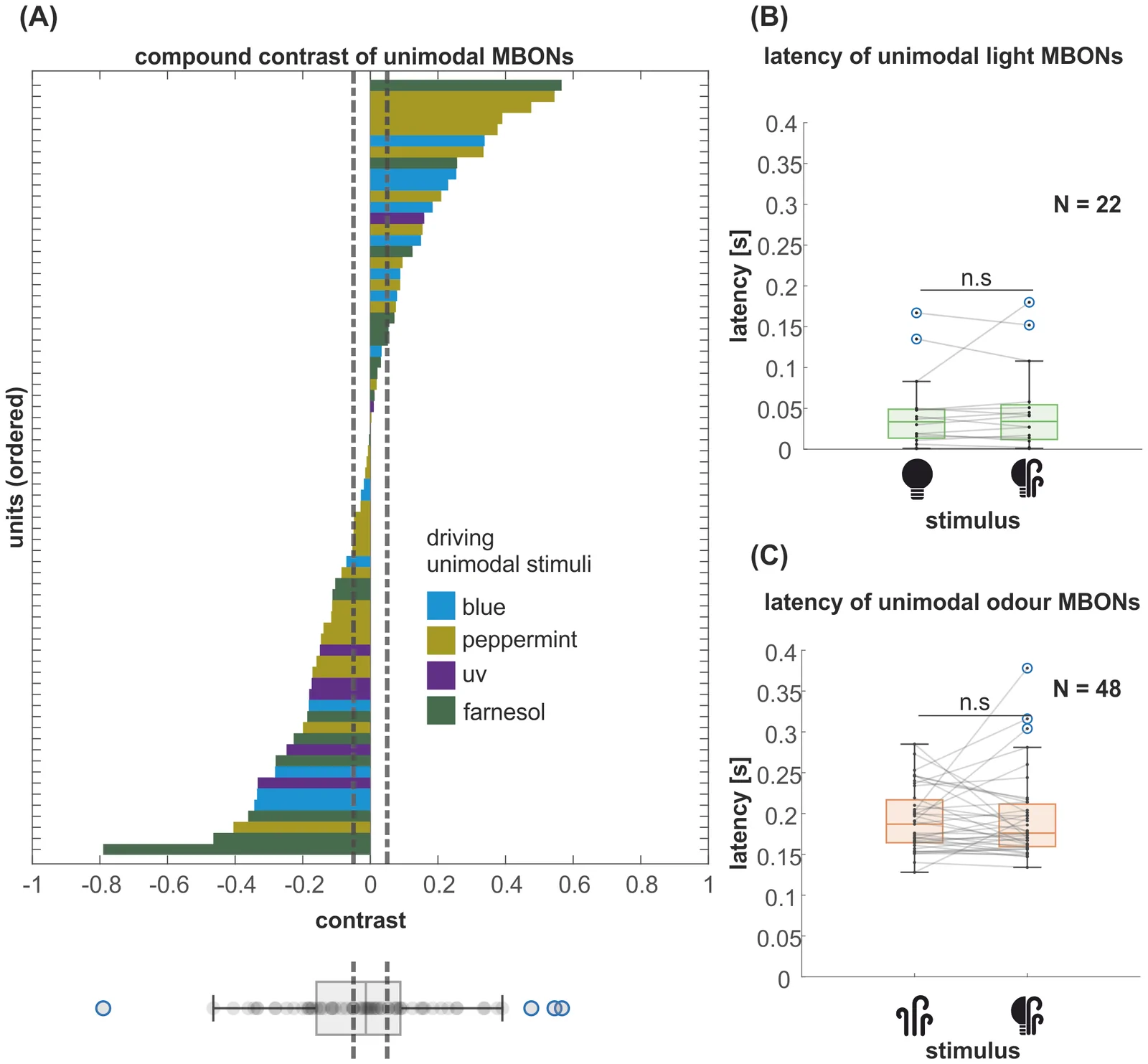
Compound-mediated modulation in unimodal MBONs: **(A)** Compound contrasts of the unimodal MBONs. Each bar is the contrast of a single unimodal MBON representing the difference between the response strength induced by the unimodal element (odour or light) and the response strength induced by the odour-light compound. Colours indicate the unimodal element that activated the corresponding MBON. The vertical dashed lines mark contrast values of -0.05 and +0.05, respectively. The boxplot below illustrates the distribution of the contrasts. **(B)** Latencies of light- and compound-induced responses in unimodal light MBONs (Friedman test, p=0.3711; n.s.) and **(C)** latencies of odour- and compound-induced responses in unimodal odour MBONs (Friedman test, p= 0.1489; n.s.) were not different.

Again, *A* is the AUC of the positive rate response to the compound (C), light (L), and odour (O), respectively. The closer this contrast value is to 0, the more similar the compound response is to the unimodal response. A value > 0 suggests that the compound response was “enhanced” by the presence of the other modality, while a value< 0 suggests that the compound response was “suppressed” by the presence of the other modality (example shown in **Figure 2E**). The magnitude of the contrast values indicated the extent of suppression or enhancement (**Figure 5A**).

### 2.10 Modelling

**Figure 6:**
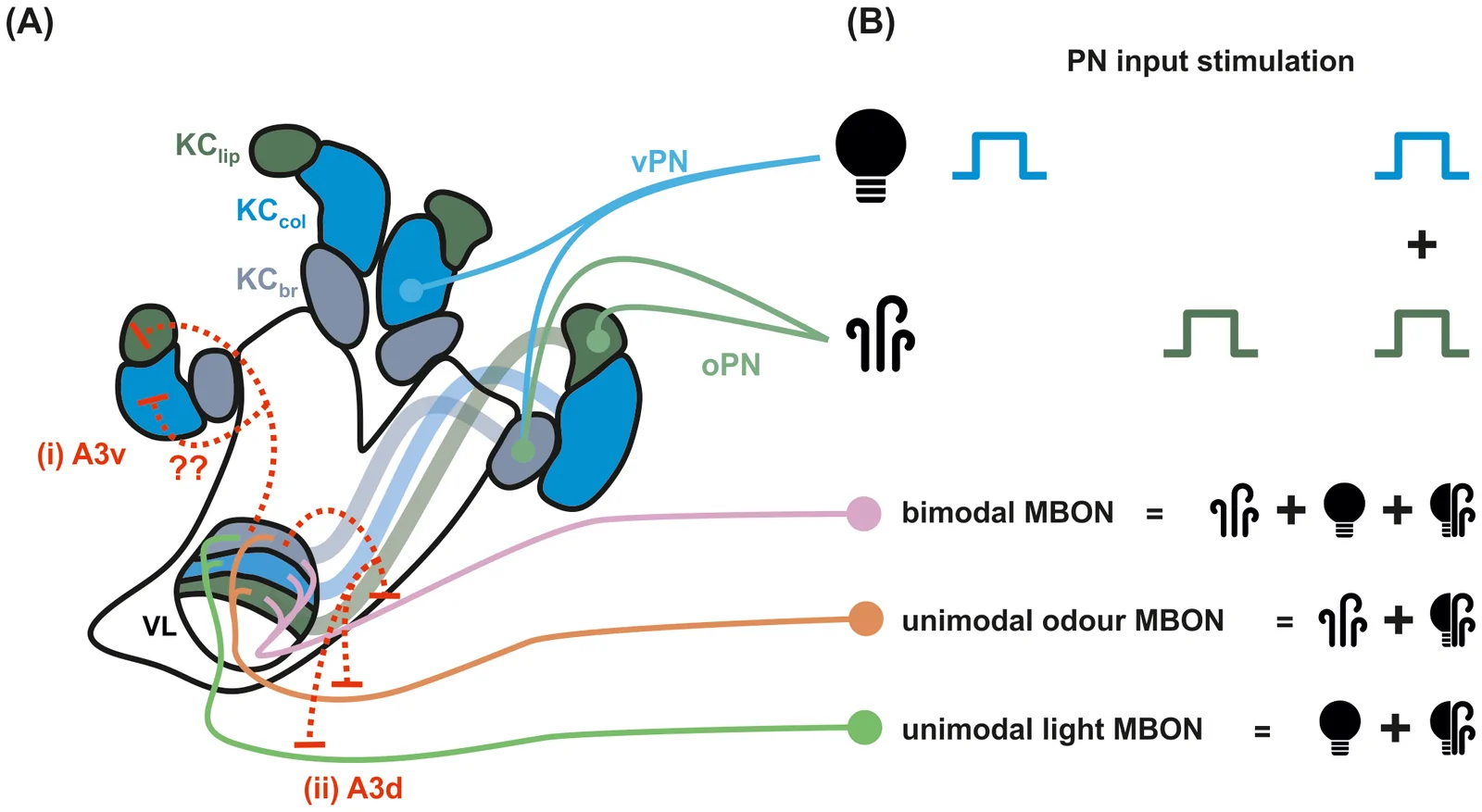
Model of the mushroom body circuit: **(A)** Circuit model of the mushroom body (adapted from Groh and Rössler, 2020, licensed under CC BY 4.0), including all engaged connections. Olfactory projection neurons (oPN) innervate Kenyon cells (KC) of the lip (*KC_lip_*), and basal ring (*KC_br_*), while visual projection neurons (vPN) innervate the collar (*KC_col_*), and basal ring KCs. The three KC axon types (based on the input region) run in parallel to distinct layers of VL, where they connect to the different MBON types, carrying light, odour, and compound information. In addition, we included two possibilities of morphologically described inhibitory connections by neurons of the GABAergic A3 cluster. (i) A3v-KC feedback inhibitions, where A3v neurons relay information from the VL *KC_br_* inhibiting the *KC_lip_* (observed by Grünewald, 1999a, b; Zwaka et al., 2018) and *KC_col_* (not observed so far) and (ii) parallel A3d-MBON feedforward inhibitions, where A3d neurons relay information from VL *KC_br_* inhibiting the three MBON phenotypes locally within the VL and medial lobe. **(B)** *Top*: Simulation protocol for replicating light, odour and compound stimuli in the experiment by exciting the corresponding PNs with a step function. *Bottom* In the VL, unimodal odour MBONs are connected to *KC_lip_* and *KC_br_*. Unimodal light MBONs receive input from *KC_col_* and *KC_br_*. Bimodal MBONs receive input from all three KC types.

We used coupled ordinary differential equations to build a rate propagation model in MATLAB. Starting with the oPNs and vPNs up to the MBONs, each involved neuronal layer (**Figure 6A**) was simulated by its own equation. The equations were solved simultaneously at each time step using the Euler method with a small time step (dt) of 0.001 s. According to the recording protocol, a stimulus was presented in silico as a step input to the related pathway, lasting 3s (**Figure 6B**). A visual stimulus activated the vPNs, an odour stimulus activated the oPNs, and a compound stimulus activated both vPNs and oPNs simultaneously.

#### 2.10.1 Projection neurons

Both PN types were simulated as adaptive leaky integrators (Brette and Gerstner, 2005) responses to the step stimulus, as observed in a prior extracellular study (Strube-Bloss et al., 2012) and modelled in the following way (Equations 1, 2) (**Figure 6B**):

(1) $$\frac{dPN}{dt}=-w_{r}PN+w_{i}\left(I-a\right)$$

(2) $$\frac{da}{dt}=-ka+w_{a}PN$$

Where *PN* is the neuronal rate response of either oPNs or vPNs, and *a* is the adaptation variable of the leaky integrator, which is used as a hidden variable to replicate the spike rate adaptation of a phasic-tonic response (supplemental **Supplementary Figures 2A–C**). Physiologically, the adaptation term may capture the proportion of the potassium current in PNs, as reported in a patch-clamp study (Kropf and Rössler, 2018). *I* is the step input function, and *w* are the weight parameters; k (value = 1) is for dimensional consistency.

#### 2.10.2 Kenyon cells

Based on their morphologically described innervation patterns, KCs can be divided into three populations: lip KCs (*KC_lip_* for olfaction), collar KCs (*KC_col_* for vision) and basal ring KCs (*KC_br_* for compound). Temporal sparseness in the KC layer mostly follows high-pass filter properties (Szyszka et al., 2005) and may be implemented by KC input as a function of PN rate derivative. The oPNs are connected to *KC_lip_* and *KC_br_*, while vPNs are connected to the *KC_col_* and *KC_br_*. The *KC_br_* encodes compound stimuli by multiplying the derivatives of the vPN and oPN rates as its inputs (**Figure 6A**). This simplification allows it to convey the compound stimulus information (supplemental **Supplementary Figures 2D–F**). The corresponding equations (Equations 3–5) are:

(3) $$\frac{dKC_{lip}}{dt}=-w_{l}KC_{lip}+w_{o}\frac{dPN_{o}}{dt}-w_{n}A_{3v},$$

(4) $$\frac{dKC_{col}}{dt}=-w_{c}KC_{col}+w_{v}\frac{dPN_{v}}{dt}-w_{n}A_{3v},$$

(5) $$\frac{dKC_{br}}{dt}=-w_{br}KC_{br}+w_{c}\frac{dPN_{v}}{dt}\frac{dPN_{o}}{dt},$$

where *KC* is the neuronal rate response of different KC subpopulations, *w* are the associated weights, and *A*_3_*_v_* is the neuronal rate response of A3v neurons (only present during the feedback inhibition case).

#### 2.10.3 GABAergic inhibitory feedback neurons of the A3 cluster

**Figure 7:**
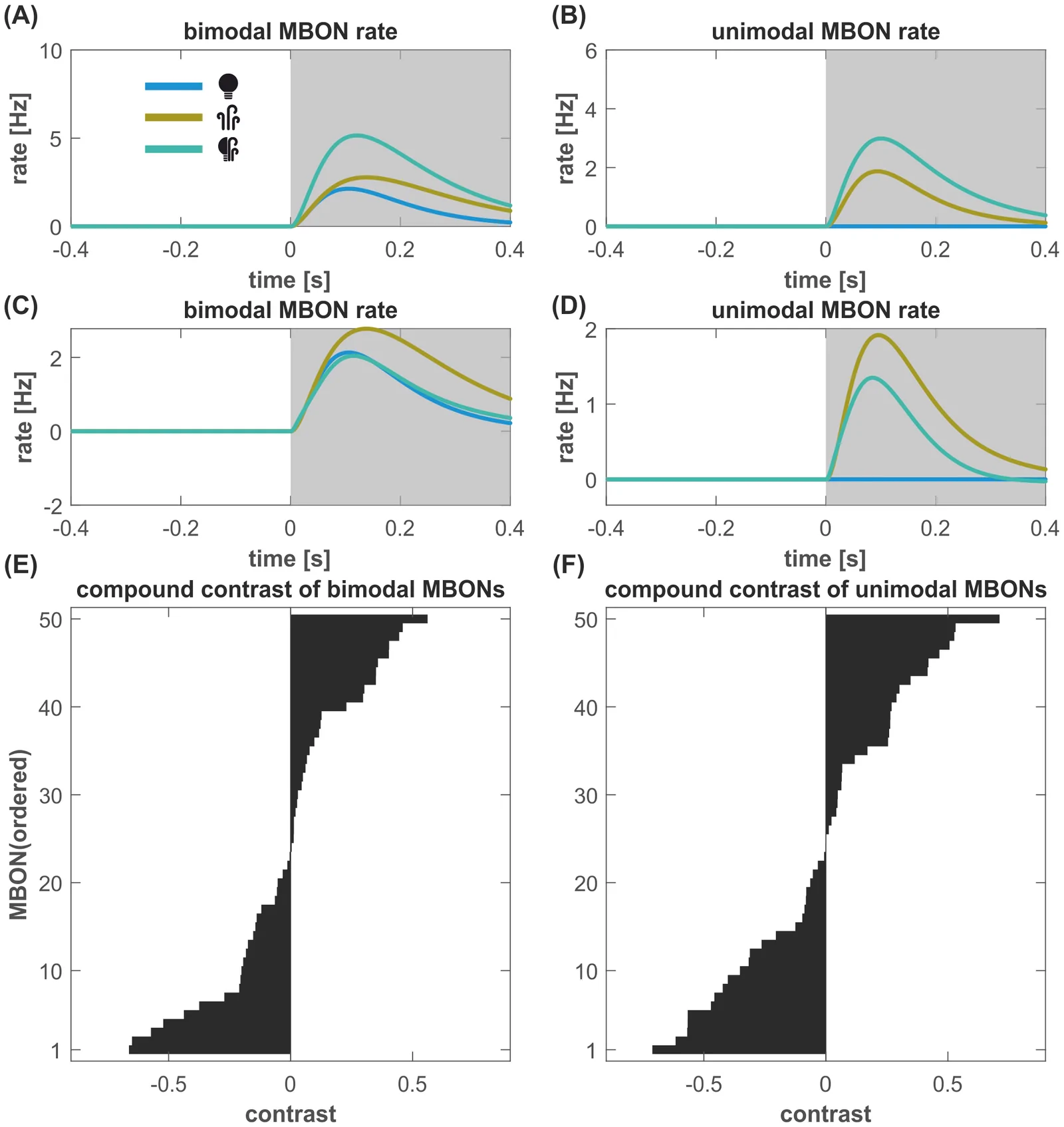
Rate propagation model with compound pathway (*KC_br_*) and A3d-MBON inhibition. **(A)** A simulated bimodal MBON. **(B)** A simulated unimodal odour MBON. Both were connected to the exciting compound and the inhibitory parallel A3d-MBON pathway. In both MBON subgroups, the simulated rate response to the compound was enhanced compared to the odour or light responses if the weights of the compound pathway were bigger than the weights of the A3d-MBON inhibition. **(C, D)** Same as in A and B, but the weights of the compound pathway were less than the inhibitory connections, resulting in a compound-mediated suppression. **(E)** Contrasts for a population of 50 simulated bimodal MBONs replicating both response phenotypes shown in A and **(C)** The variability in contrasts was generated by the relative strengths of the excitatory compound pathway and the inhibitory A3d connections. **(F)** Same as in E, but for unimodal MBONs.

**Figure 8:**
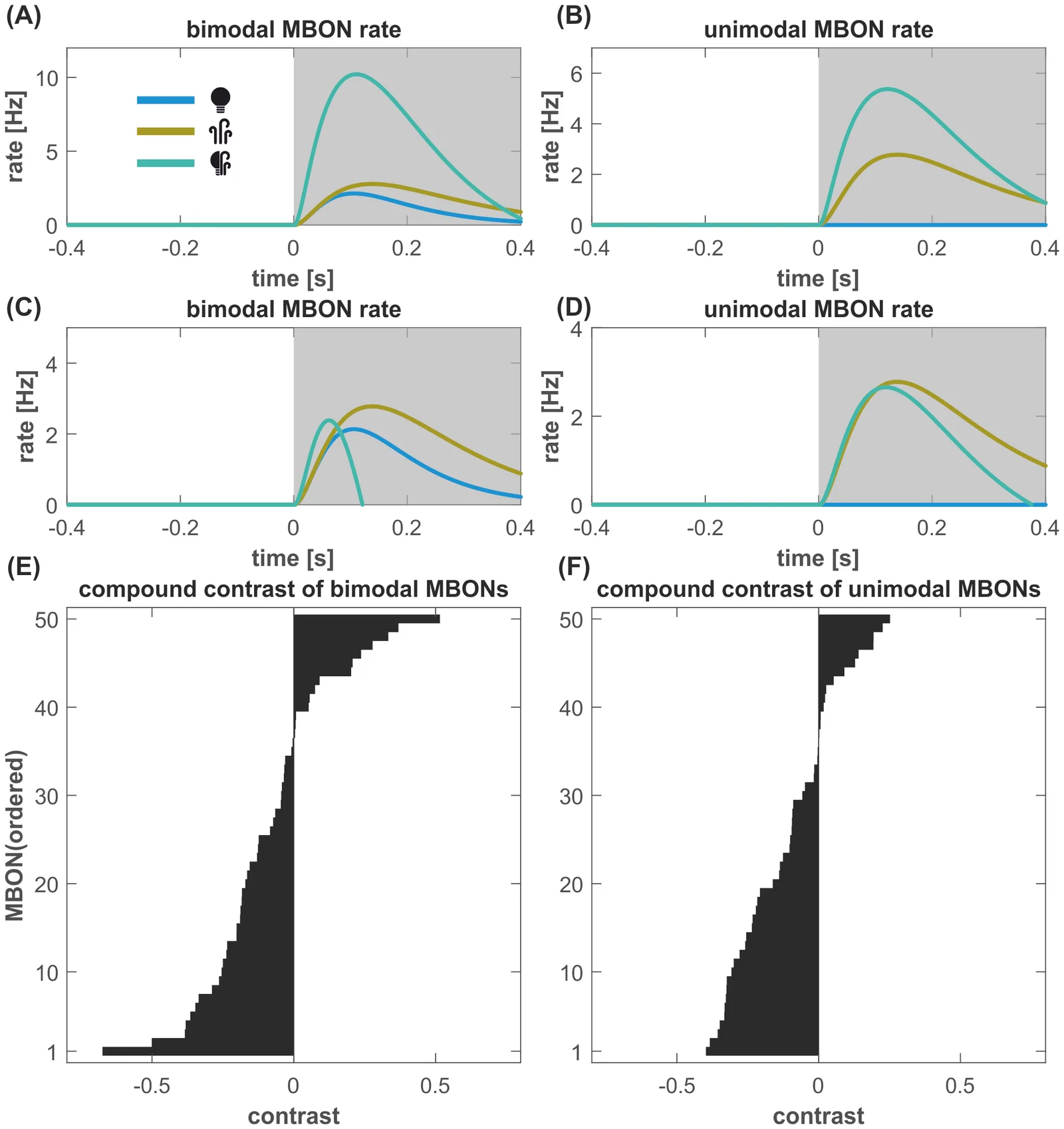
Rate propagation model with compound pathway (*KC_br_*) and A3v-KC inhibition **(A)** A simulated bimodal MBON. **(B)** A simulated unimodal odour MBON. Both were connected to the exciting compound and the inhibitory A3v-KC feedback pathway. In both the MBONs, the simulated rate response to the compound was enhanced compared to the odour or light responses if the weights of the compound pathway were bigger than the weights of the A3v-KC inhibition. **(C, D)** Same as in A and B, but the weights of the compound pathway are less than the inhibitory connections (less excitation, more inhibition), resulting in a compound-mediated suppression. **(E)** Contrasts for a population of 50 simulated bimodal MBONs replicating both response phenotypes shown in A and **(C)** The variability in contrasts was generated by the relative strengths of the excitatory compound pathway and the inhibitory A3v connections. **(F)** Same as in E but for unimodal MBONs.

Following their morphology, A3 neurons are subdivided into two inhibitory pathways (explored one at a time in separate circuit models, **Figures 7**, **8**); (i) A3v neurons receiving inputs from *KC_br_* and innervating the *KC_lip_*/*KC_col_* giving proper inhibitory feedback to the overall MB circuit, or (ii) A3d neurons receiving input from basal ring KCs, inhibiting different MBONs, making the MB circuit a parallel feedforward circuit with inhibition at the very end. The equations (Equations 6, 7) are:

(6) $$\frac{dA_{3v}}{dt}=-w_{a3}A_{3v}+w_{a_{br}}KC_{br},$$

(7) $$\frac{dA_{3d}}{dt}=-w_{a3}A_{3d}+w_{a_{br}}KC_{br},$$

where *A*_3_*_v_*, *A*_3_*_d_* are the neuronal rate responses of the corresponding A3 neuron clusters and *w* are the associated weights.

#### 2.10.4 Mushroom body output neurons

Bimodal MBONs were activated by *KC_lip_*, *KC_col_* and *KC_br_*, while unimodal odour MBONs were connected to *KC_lip_* and *KC_br_* only. They receive inhibitory input from A3d neurons (part of the parallel feedforward inhibition circuit) that, in turn, receive input from *KC_br_* giving rise to the suppressive functional phenotypes. Similar connectivity logic applies to unimodal MBONs, where unimodal light MBONs receive input from *KC_col_* and *KC_br_* and unimodal odour MBONs from *KC_lip_* and *KC_br_* (**Figure 6B**). The corresponding equations (Equations 8–10) are:

(8) $$\frac{dMBON_{bi}}{dt}=-w_{1}MBON_{bi}+w_{2}KC_{lip}+\;w_{3}KC_{col}+w_{4}KC_{br}-w_{n}A_{3d},$$

(9) $$\frac{dMBON_{l}}{dt}=-w_{5}MBON_{l}+w_{6}KC_{col}+\;w_{7}KC_{br}-w_{n}A_{3d},$$

(10) $$\frac{dMBON_{o}}{dt}=-w_{8}MBON_{o}+w_{9}KC_{lip}+\;w_{10}KC_{br}-w_{n}A_{3d},$$

where *MBON* is the neuronal rate response of different MBON subpopulations, *w* are the associated weights, and *A*_3_*_d_* is the neuronal rate response of A3d neurons (only present during the parallel feedforward inhibition case).

## 3 Results

### 3.1 Categorisation of uni- and multimodal mushroom body output neurons based on response patterns

The population of 140 potential MBONs recorded from 43 bees (**Figure 3A**) shows that the separation of modalities at the MB input side is partially conserved and partially not. Some MBONs responded to only one modality, while others responded to both. Most of the responses are of a phasic character with peaks at the onset and in some cases also at the offset of a stimulus. We neglect the latter in our analyses, since we are interested in the initial response to the uni- and bimodal stimuli. In addition to the phasic activity, a few neurons also showed tonic activity, which seemed to be more prominent when the stimulation included an odour element. By setting a threshold of 5xσ, we translated the heatmap of the mean firing rates of each unit (**Figure 3A**) into a binary response matrix (**Figure 3B**). Overall, we found different combinations (**Figure 3C**). Some units responded to only one or both wavelengths and were categorised as unimodal light MBONs (20%). MBONs sensitive to one or both of the odours were categorised as unimodal odour MBONs (34%). A third category responded to both modalities and was defined as bimodal MBONs (11%). Furthermore, a small subpopulation of 7 MBONs was found to be activated only when two of the modalities were presented together, which could be the result of sub-threshold summation. However, since we were interested in cross-modal effects in MBONs responding to at least one single modality and a compound stimulus that included that stimulus element, we excluded these 7 MBONs from our further analysis.

### 3.2 Suppression and enhancement during bimodal integration

The contrast between the sum of the response strengths induced by the unimodal elements (odour plus light) and the response strength induced by the compound showed that in some bimodal MBONs, the compound induced a higher response compared to the sum (enhancement). Meanwhile, in others, the compound-induced activity was lower than the sum (suppression). This was quantified using an additivity contrast. A contrast close to or at “0” would reflect the linear summation of the response strengths of the unimodal elements during compound stimulation. This occurred rather rarely (**Figure 4A**). Although the response strength of the compound could be modulated in favour of one modality or the other, the response latency was dominated by the visual element of the compound. Comparing the latencies of the olfactory-visual compound with the latencies induced by the single elements revealed that the fast light response dominated the compound-induced latency, since there was no difference between compound and light (p value: 0.6273, *post-hoc* test: Tukey-Kramer Test), but a significantly slower response was induced by the odour stimulus (p value: 0.0005, *post-hoc* test: Tukey-Kramer Test) (**Figure 4B**).

### 3.3 Parallel pathways for uni- and bimodal processing

Unimodal MBONs included two subpopulations: unimodal odour and unimodal light MBONs. In addition to their unimodal responses, both groups also responded to the olfactory-visual compound presentation. The response to the compound was not the same as the one induced by the unimodal element to which the MBON was sensitive, even though the same concentration (odour) or intensity (light) was used. Instead, stimulation with an olfactory-visual compound modulated the response strength to the unimodal element for which the unit was sensitive. As in the case of the bimodal MBONs, some units showed a higher contrast compared to the single modality, whereas others showed a lower contrast (**Figure 5A**). Seventeen of the unimodal MBONs were affected rather slightly, showing contrasts between -0.05 and +0.05 (**Figure 5A**). This suggests that these units may mainly respond to the light or odour element during compound presentation. However, a major proportion of 29 unimodal MBONs showed a decreased response strength when stimulated with the olfactory-visual compound, with contrasts ranging from -0.05 to -0.79. Almost the same proportion of unimodal MBONs (N = 24) showed an increased response strength when stimulated with the compound, with contrasts ranging from 0.05 to 0.57 (**Figure 5A**). Thus, compound stimulation may activate a parallel neural pathway, which is modulating the response strength induced within the unimodal pathway by adding excitation or inhibition.

Comparing the latencies of the unimodal MBONs revealed that the modality that activated the MBON also dominated the response latency of the compound (**Figures 5B, C**). Thus, although light responses were generally faster (**Figure 5B**), the visual element did not speed up the activity induced by the olfactory-visual compound in unimodal odour MBONs (**Figure 5C**).

### 3.4 Replicating functional MBON responses using a rate propagation model

Starting with the oPNs and vPNs, we built a rate propagation model that included a KC layer, divided in KCs of either the lip, the collar, or the basal ring, which activated an MBON layer with different MBON subpopulations (**Figure 6**). The model follows the circuit connections and the overall response dynamics at each layer, i.e., the phasic-tonic responses in the PN layer, the temporal sparseness in the KC layer (Supplemental **Supplementary Figure 2**), and the phasic responses in the MBON layer (**Figures 7A–D**, **8A–D**). The contrast analysis of the recorded bimodal and unimodal MBONs (**Figures 4A**, **5A**) revealed two possible main features in the MB circuitry: (i) the existence of a parallel compound pathway and (ii) besides excitatory, also inhibitory connections associated with that pathway. The compound pathway was implemented within the basal ring KCs by multiplying the light and odour inputs, showing compound-specific responses (Supplemental **Supplementary Figure 2F**). To model the observed compound-mediated suppression in bimodal and unimodal MBONs, we included A3 neurons, since they are known to be GABAergic (Bicker et al., 1985; Grunewald, 1999a; Zwaka et al., 2018). A3 neurons have two morphologically described target sites: the first ones connect the MB output region (VL) retrogradely with the MB calyx, known as A3v neurons (Zwaka et al., 2018). Our model implemented this as A3v-KC feedback connections. The second ones target other MBONs within and across the VL and medial lobe (A3d neurons; Zwaka et al., 2018). This is implemented as A3d-MBON parallel feedforward inhibition (**Figure 6A**). Rate responses of both unimodal and bimodal MBONs were simulated by stimulating different PN pathways alone or together with a step input (**Figure 6B**).

### 3.5 Implementing nonlinear enhanced compound responses

If the response to the olfactory-visual compound stimulus in bimodal MBONs were the sum of its unimodal elements, we would observe contrasts at or at least very close to 0. However, that was rarely the case. Instead, we observed many bimodal MBONs showing an increased contrast (positive contrasts in **Figure 4A**), suggesting that the compound adds some activity to the sum of the unimodal elements. This addition of a compound-induced activity became more apparent in unimodal MBONs, which responded to only one unimodal element, but also showed increased activity when stimulated with the compound (positive contrasts in **Figure 5A**). This suggests that a parallel excitatory pathway was activated by the compound stimulation. We implemented this into our model by connecting both unimodal and bimodal MBONs with basal ring KCs (**Figure 6A**).

Since our model has 25 parameters (supplemental **Supplementary Table S1**), we first manually tuned it to preserve qualitative features, such as phasic responses in the MBON, and then kept all other weights constant. Among these weights, we selected 4 that have the strongest impact, leading to enhancement and suppression effects (weight parameters are available in supplemental **Supplementary Table S1**). If the relative contribution from the compound pathway was greater than that of the inhibitory pathway, it could lead to the enhancement effects, i.e. the response to the compound is stronger than to the unimodal stimuli in both the unimodal and bimodal MBON (**Figures 7A, B**, **8A, B**). The weights for this were *w_c_* and *w_n_*. Since the weights were very sensitive to the range and order of magnitude, we randomised the weights while preserving the qualitative features. For the contrasts in the bimodal MBON (top right side of **Figure 7E**), *w_c_* was a random value drawn from the range 0 to 1, multiplied by the factor 10^-3^. The weight *w_n_* was a random integer ranging from 1 to 200. In addition, to account for variability in light and odour responses, we also chose a random integer from 0 to 1 for the corresponding weights ( $w_{a_{vpn}}$, $w_{a_{opn}}$). The contrasts were calculated in the same way as for the bimodal and unimodal contrasts in the experimental data. Since the model does not account for delay/latency in the responses, we calculated the AUC as the positive area from 0 to 0.4s. Note that the randomisation process could create both positive and negative contrasts. In total, we simulated 50 bimodal and unimodal MBONs and their contrasts using this randomisation process. In the unimodal MBON cases, we showed only examples of unimodal odour MBONs (**Figures 7B, D, F**, **8B, D, F**), but the results hold true for both unimodal light and odour MBONs, as both pathways (light and odour) are equivalent in the model.

### 3.6 A3d-MBON parallel inhibition can lead to compound-mediated suppression

The contrast analysis of the MBONs shows negative contrast in both bimodal and unimodal MBONs (**Figures 3A**, **4A**), supporting the existence of compound-mediated inhibition. In the A3d-MBON parallel feedforward inhibition, A3d neurons were connected to the compound pathway and inhibited all three MBON types. Here, the MBONs integrated the compound pathway and the inhibitory connection within the same layer, leading to suppression of the compound responses relative to the unimodal responses. The compound response, even though suppressed, qualitatively resembled the unimodal response (**Figures 7C, D**). The strength of the negative contrast depends on the relative strength between the compound pathway and the inhibitory feedback at the MBONs. To generate variation, we used the same randomisation technique as mentioned above, using the same variables, which were *w_c_*, $w_{a_{vpn}}$, $w_{a_{opn}}$ and *w_n_* in both the bimodal and unimodal MBONs (**Figures 7E, F**).

### 3.7 A3v-KC feedback inhibition can lead to compound-mediated suppression

Another inhibition mechanism was introduced as feedback at the KC level. In the A3v-KC feedback, A3v neurons are also connected to the compound pathway, inhibiting either the collar or the lip KCs. Here, to obtain the negative contrast in the MBONs, the relative contribution from the compound pathway had to be less than that from the inhibitory pathway. As this feedback occurred at an earlier processing stage (the KC layer), the compound response looked qualitatively different from the light/odour responses (**Figures 8C, D**). Since this inhibition was different here, the model has to be tuned separately from the KC level. We use similar parameters (compared to the feedforward inhibition case) *w_c_* for the compound pathway was set to a random value drawn from the range of 0 to 1 multiplied by 10^-3^, and *w_n_* was set to a random integer between 10 and 200 (**Figure 8E**). In this case, the *w_n_* acts as both the inhibitory connection strength and the light, odour response variation. For the bimodal contrast variations, we used the same criteria for *w_c_*, *w_n_*. However, we used an additional weight (*w*_4_) of the compound pathway (*KC_br_*) as input into the bimodal MBONs and set it to a random integer between 1 and 50 (**Figure 8F**). Note that the response strength varies when comparing the enhancement effects of the A3v-KC feedback and the A3d-MBON model (**Figures 7A, B**, **8A, B**). This likely arises due to the tuning differences that are necessary when connections differ, but the qualitative features of the response must be preserved. This does not imply that one model is better than another.

## 4 Discussion

To understand multisensory integration, we recorded MBONs in live honeybees and analysed their responses to two odours, two lights, and two odour-light compound stimuli. In honeybees, seven anatomically defined groups of MBONs have been described (Rybak and Menzel, 1993; Strausfeld, 2002; Kirschner et al., 2006; Zwaka et al., 2018). These groups differ in the locations of their cell bodies, there depth of axodendritic arborisation, and their branching patterns within the MB lobes and outside there (Rybak and Menzel, 1993), suggesting that the different groups may have different functions. Indeed, we found distinct MBON response profiles that might be related to different clusters. Based on the depths of our recordings (**Supplementary Table S2** and **Supplementary Figure 3**), we speculate that the data may sample more of the A1, A2, A4, and A7 clusters, as these units integrate from the ventral part of the vertical lobe at average depths of 115-125 µm (A1 and A2), 70-90 µm(A4), 90 µm (A7) respectively (Rybak and Menzel, 1993).

We were able to categorise unimodal odour, unimodal light, and bimodal MBONs (**Figure 3C**), replicating our earlier findings, which we further analysed here to understand cross-modal modulatory effects. Although the proportion of the different types of MBONs varies compared to our previous study (Strube-Bloss and Rössler, 2018), which we interpreted as a bias since the datasets were recorded by different experimenters, both preferring a slightly different recording positions and depths (cp. **Supplementary Table S2**; **Supplementary Figure 3**), we could reproduce the different uni- and multi- modal responding MBON types.

Furthermore, we found a small population of seven MBONs that were only activated when both modalities occurred together, which could be interpreted as the result of sub-threshold summation, and a larger group of MBONs (30%) that did not respond at all to our stimulus set. The latter might have the capacity to become recruited during a classical conditioning experiment to encode a stimulus-reward association, as reported in our earlier studies (Strube-Bloss et al., 2011, 2016). The phasic responses we recorded here are typical for MBONs, as supported by intracellular recordings of the A3 cluster (Grünewald, 1999b), and are most likely the result of temporally sparse KC activity (Szyszka et al., 2005; Ito et al., 2008) driving these MBONs.

### 4.1 A parallel olfactory-visual pathway modulates unimodal processing

At the MB input side, olfactory, visual, and olfactory-visual information is organised in a topographical way, innervating distinct calycal layers (Gronenberg, 2001; Groh and Rössler, 2020; Habenstein et al., 2023). The lip is innervated by oPNs, whereas the collar receives visual input from vPNs. Only the basal ring is innervated by both modalities. Looking at the MB output side, this topological organisation separating modalities seems to be conserved. This was reflected most drastically in the response profiles of unimodal MBONs, which are not activated by a second modality. However, the response rate to the unimodal element of both types of unimodal MBONs was modulated when stimulated with the olfactory-visual compound, reflected in an up- or down-regulation (**Figure 5A**). In addition, the latency analysis shows that the compound responses are as fast as the fastest modality (in the bimodal MBON case, **Figure 4B**) or equal to that of the responding unimodal stimuli (in the unimodal MBON case, **Figures 5B, C**), suggesting a temporal additive effect in the MB circuit. To produce such a response profile, the compound has to activate an additional neural pathway that responds only when stimulated with the compound. The basal ring is most likely the neural substrate and the starting point of such a parallel multimodal pathway, as it is the first processing level at which both modalities (olfaction and vision) converge within the same layer (Gronenberg, 2001; Groh and Rössler, 2020; Habenstein et al., 2023). Although a final proof of convergent synapses within the basal ring is still lacking ultrastructural analysis, unlike the lip and collar (Nicolaidou et al., 2026), further studies are needed to confirm this. Following our recorded response profiles, we argue that unimodal light MBONs receive input from *KC_col_* and *KC_br_*, whereas unimodal odour MBONs are innervated by *KC_lip_* and *KC_br_*. Thus, the compound pathway via the basal ring innervates both unimodal MBON populations, modulating their neural activity and was therefore implemented in our model in a parallel way (**Figure 6**). Most interestingly, the character of this modulation we observed was not stereotypic, meaning that the response strength to the unimodal element was sometimes enhanced and sometimes suppressed, suggesting the activation of excitatory and inhibitory neurons, respectively, contributing to the compound-induced neural activity. Multi-site imaging experiments at the MB (Carcaud et al., 2023) with multimodal stimuli could be a way to explore this possibility further. If no regions (basal ring or even other regions) activate to the compound-specific stimuli, that would suggest that these response patterns might be the result of MBON level computations rather than a dedicated compound pathway.

### 4.2 Compound-mediated enhanced responses in MBONs

According to our contrast measure, a linear summation of the recorded responses induced by the light and odour pathways in bimodal MBONs would yield a contrast of zero. Instead, we observed a non-linear additive effect resulting in contrasts greater than zero (**Figure 4A**). This becomes more evident in unimodal MBONs, which did not respond to the second element, odour or light, respectively, but showed increased activity to the compound presentation (**Figure 5A**). As mentioned above, this suggests that the compound might activate an additional excitatory neural pathway.

Our model implements such a pathway at two processing levels. At the KC level, KCs connecting to this pathway carry compound information (Supplemental **Supplementary Figure 2F**) and simply multiply the modalities.

At the MBON level, unimodal and bimodal MBONs receiving excitatory input from the *KC_br_* also lead to positive contrasts (positive values in **Figures 7E, F**, **8E, F**). Although not explored in this paper, this compound-mediated enhancement might be a neuronal circuit mechanism underlying elemental learning, such as positive patterning in honeybees, where only the compound was associated with the reward and discriminated from its single elements (Mansur et al., 2018; Becker et al., 2019; Strube-Bloss et al., 2023).

### 4.3 Feedback-inhibition within the olfactory-visual pathway

The negative contrast values observed in bimodal and unimodal MBONs when stimulated with the olfactory-visual compound (**Figures 4A**, **5A**) suggest the activation of inhibitory neurons. The GABAergic neurons of the protocerebral-calycal tract are potential candidates providing the necessary suppression (Grünewald, 1999a). They belong to the A3 cluster (Rybak and Menzel, 1993), representing a specialised group of MBONs. About half of the A3 neurons (A3v) send retrograde feedback from the MB output to its own input, making connections to different regions of the MB calyces (Zwaka et al., 2018; Grünewald, 1999a, b). Among these, some subgroups are capable of establishing cross-modal inhibitory connections, such as the basal ring-to-lip inhibition, suggesting that olfactory-visual information could modulate the perception of the olfactory component within the lip. However, a basal ring-to-collar connection has not been observed so far, but would explain the negative contrasts of the unimodal visual MBONs when stimulated with the compound. When we implemented such a connection within our model, we could reproduce the olfactory-visual compound-induced responses in unimodal visual MBONs (**Figures 8D, F**), leading to the hypothesis that such a connection might exist and should be further explored by ultrastructural analysis. Other studies have shown that A3v neurons are plastic (Grünewald, 1999b; Haehnel and Menzel, 2010), most likely by receiving input from the Ventral Unpaired Median neuron of the Maxillary neuromere number 1 (VUMmx1), facilitating the reward pathway (Hammer, 1993; Sinakevitch et al., 2011). Studies using GABA blockers at the MB calyces have shown the importance of A3v in reversal learning of odours, but may not be important in odour discrimination (Boitard et al., 2015). However, the convergence of the olfactory-visual pathway via *KC_br_*, A3v, and the VUMmx1 could serve as a neural substrate to solve olfactory-visual learning tasks, as mentioned above.

### 4.4 Parallel feedforward inhibition at the MBON level

A second group of inhibitory neurons in the A3 cluster are the lobe-connecting A3d neurons, which could provide connections within and across the MB output lobes (Zwaka et al., 2018; Grünewald, 1999a; Okada et al., 2007). The model predicts that such inhibitory connections preserve a unimodal response pattern because the excitation from the compound pathway and the inhibition from these connections are integrated by the MBON itself, removing any temporal distortions, as observed in **Figures 8C, D**. While inhibition at the MB calyx provides proper feedback, which could make the network slightly more energy-efficient and stable, parallel feed-forward inhibition at the MBON level may preserve the temporal properties of the layers above. However, both subpopulations of A3 neurons exist in parallel, and our model shows that for compound-mediated suppression, either inhibitory connection could suffice. One way in which both types of neurons could be optimally used would be by A3v providing an overall gain control and A3d providing fine-scale modulation, which could be explored by further developing our model. Even though we could predict the response patterns with a few parameters (cp. **Supplementary Table 1**), we have not fully explored the sensitivity and role of every parameter.

From our models, it is hard to conclude which one is the better model due to the lack of intermediate neuronal responses like those of the PNs and KCs. However, the models suggest that a compound pathway and connected inhibitory connections would be sufficient to produce the response profiles we observed in the data. Thus, our model provides expanded and helpful insight into olfactory-visual sensory integration and how it is functionally included within the morphologically described MB circuitry, including feedforward excitation, inhibition, and feedback inhibition.

## Data Availability

The raw data supporting the conclusions of this article will be made available by the authors, without undue reservation.
