# Supplementary material for: Form informs function: innervation patterns of projection neurons correspond to response profiles of mushroom body output neurons during olfactory-visual integration
Source: Front Physiol. 2026 Aug 18;17:1898182. doi: 10.3389/fphys.2026.1898182 (PMC13531775; doi:10.3389/fphys.2026.1898182)
Supplement: Supplementary file 1 [file DataSheet1.pdf]

Supplementary Material

Form Informs Function: Innervation Patterns of Projection Neurons Correspond to Response Profiles of Mushroom Body Output Neurons During Olfactory-Visual Integration

Athil Althaf Aliyam Veetil Zynudheen[1], Andrea Rafaela Nicolaidou[2], Claudia Groh[2], Volker Dürr[1], Wolfgang Rössler[2], Martin Strube-Bloss[1*]

[1] Biological Cybernetics, Department of Biology, Bielefeld University

[2] Behavioral Physiology and Sociobiology (Zoology II), Biocenter, University of Würzburg

* Correspondence: Martin Strube-Bloss**,** martin.strube-bloss@uni-bielefeld.de

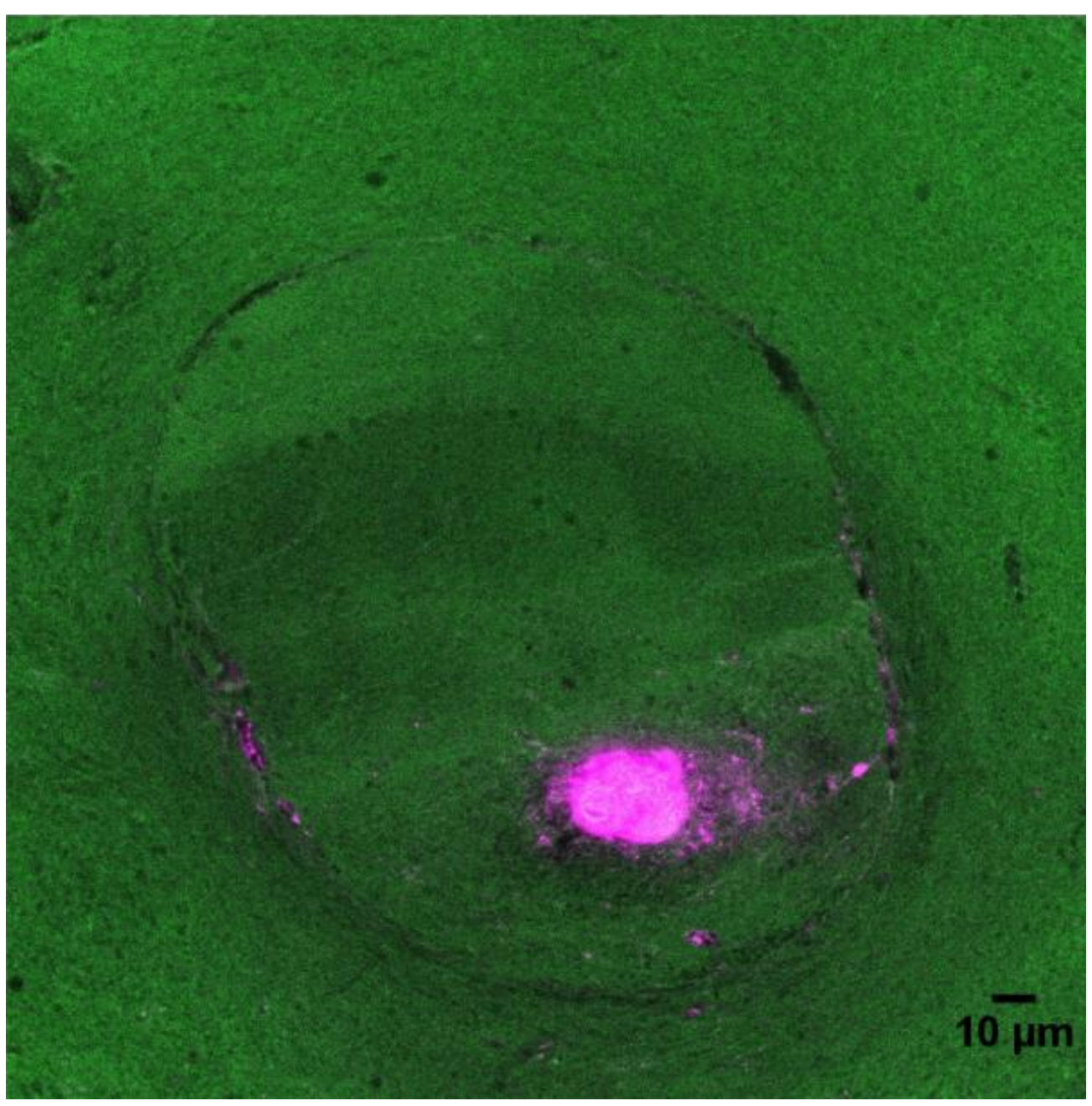

Figure S1: Electrode position: Example staining of the recording position using Alexa Fluor 647 hydrazide (magenta) at the electrode tip combined with autofluorescence (green) for background staining. The VL border and the electrode region were traced out frame by frame for the electrode reconstruction (Figure 1D).

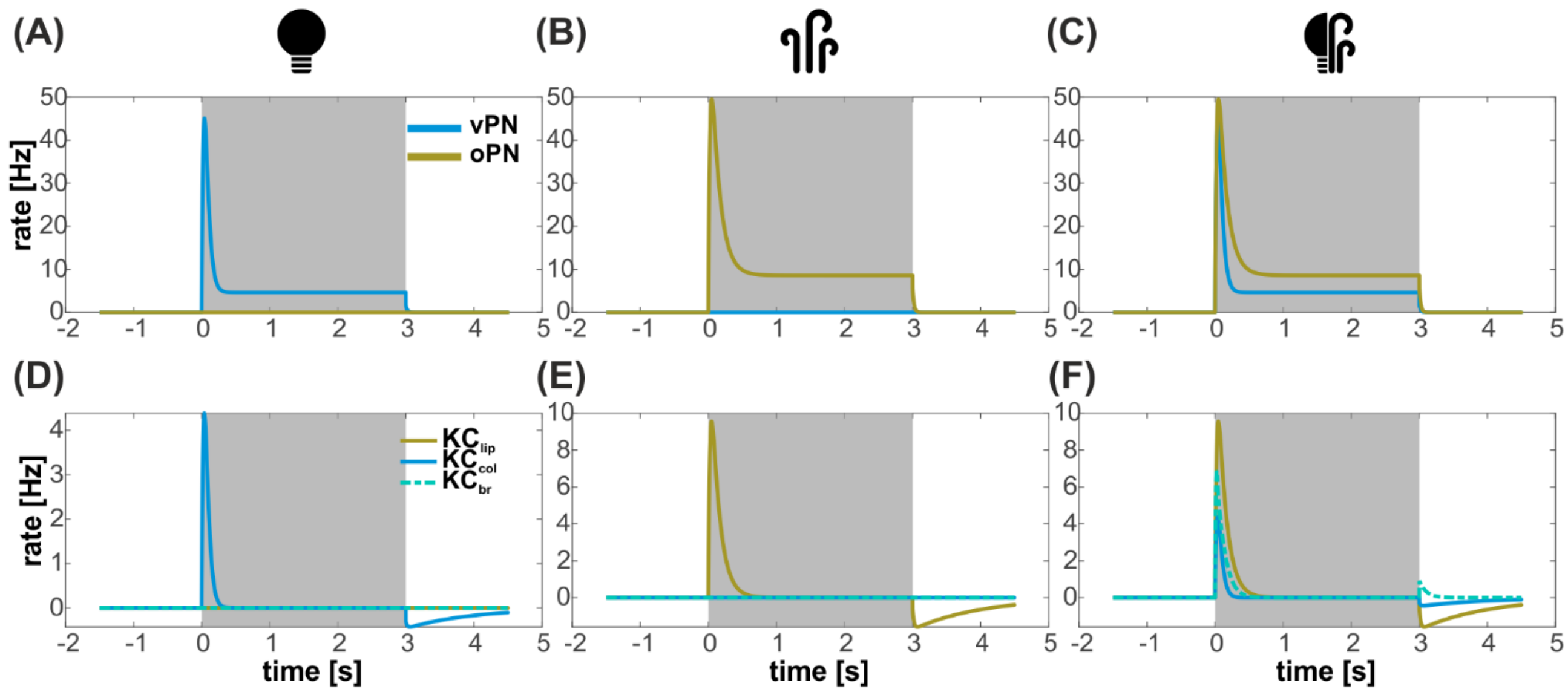

Figure S2: Rate propagation model at the PN and KC level: Rate response of different PNs when stimulated with light (**A**), odour (**B**) and compound (**C**). The vPNs are activated by light stimulation, oPNs by odour stimuli, and both are excited during compound stimulus presentation. PNs show a characteristic phasic-tonic response when activated. Rate response of different KCs that are connected to vPN (KC_{col} and KC_{br}), and oPN ($KC_{lip}$and KC_{br}) when stimulated with light (**D**), odour (**E**) and compound stimuli (**F**). Note that, even though the basal ring KCs are connected to both PN types, they only respond during the compound stimulus carrying the compound information. The KC responses represent high-pass-filtered PN responses.

#	Weight parameter (dimension)	A3d-MBON parallel feedforward inhibition (bimodal and unimodal MBON)	A3v-KC feedback inhibition (bimodal MBON)	A3v-KC feedback inhibition (unimodal MBON)
1	$w_{r_{opn}}$ (T^{-1})	50	50	50
2	$w_{r_{vpn}}$ (T^{-1})	50	50	50
3	$w_{i_{opn}}$ (T^{-1})	3000	3000	3000
4	$w_{i_{vpn}}$ (T^{-1})	3000	3000	3000
5	k (T^{-1})	1	1	1
6	$w_{a_{opn}}$ (T^{-1})	Random value from 0 to 1	0.1	0.1
7	$w_{a_{vpn}}$ (T^{-1})	Random value from 0 to 1	0.2	0.2
8	w_o (T^{-1})	0.1	0.1	0.1
9	w_l (T^{-1})	1	1	1
10	w_v (T^{-1})	0.1	0.1	0.1
11	w_c (T^{-2})	Random value from 0 to 1 multiplied by 10^{-3}	Random value from 0 to 1 multiplied by 10^{-3}	Random value from 0 to 1 multiplied by 10^{-3}
12	w_{br} (T^{-1})	10	10	10
13	$w_{a_{br}}$ (T^{-1})	10	10	10
14	w_{a3} (T^{-1})	100	100	100
15	w_1 (T^{-1})	10	10	10

**16**	$\boldsymbol{w_2}$ **(T⁻¹)**	10	10	10
**17**	$\boldsymbol{w_3}$ **(T⁻¹)**	10	10	10
**18**	$\boldsymbol{w_4}$ **(T⁻¹)**	10	Random integer from 1 to 50	100
**19**	$\boldsymbol{w_5}$ **(T⁻¹)**	10	100	100
**20**	$\boldsymbol{w_6}$ **(T⁻¹)**	10	10	10
**21**	$\boldsymbol{w_7}$ **(T⁻¹)**	10	10	10
**22**	$\boldsymbol{w_8}$ **(T⁻¹)**	10	10	10
**23**	$\boldsymbol{w_9}$ **(T⁻¹)**	10	10	10
**24**	$\boldsymbol{w_{10}}$ **(T⁻¹)**	10	10	10
**25**	$\boldsymbol{w_n}$ **(T⁻¹)**	Random integer from 1 to 200	Random integer from 10 to 200	Random integer from 10 to 200

Table S1: All weight parameters and their respective dimensions used in the different models. Most parameters are kept constant across the models, and only 5 (shaded in green) are used to generate the variation in the contrasts shown in Fig. 7E, F and Fig. 8E, F.

Bee ID	Depth	Bee ID	Depth	Bee ID	Depth	Bee ID	Depth
bee002	175.6	bee016	187.9	bee032	120.4	bee045	108.3
bee003	178.6	bee017	148.8	bee033	175.9	bee046	160.1
bee004	199.2	bee018	196.1	bee034	111.4	bee047	151.9
bee005	131.7	bee019	194.5	bee035	160.1	bee048	170.0
bee007	128.7	bee020	200.4	bee036	123.6	bee049	201.4
bee008	226.1	bee021	177.4	bee037	170.5	bee050	165.6
bee010	179.7	bee022	170.3	bee040	133.6	bee051	190.3
bee012	149.5	bee023	178.7	bee041	120.5	bee052	174.4
bee013	168.5	bee027	145.3	bee042	200.3	bee053	188.1
bee014	185.5	bee030	135.4	bee043	184.1	bee054	168.3
bee015	124.6	bee031	198.8	bee044	139.1		

Table S2: Overview of the recording depth, with a minimum of about 108.3 µm and a maximum of about 226.1 µm.

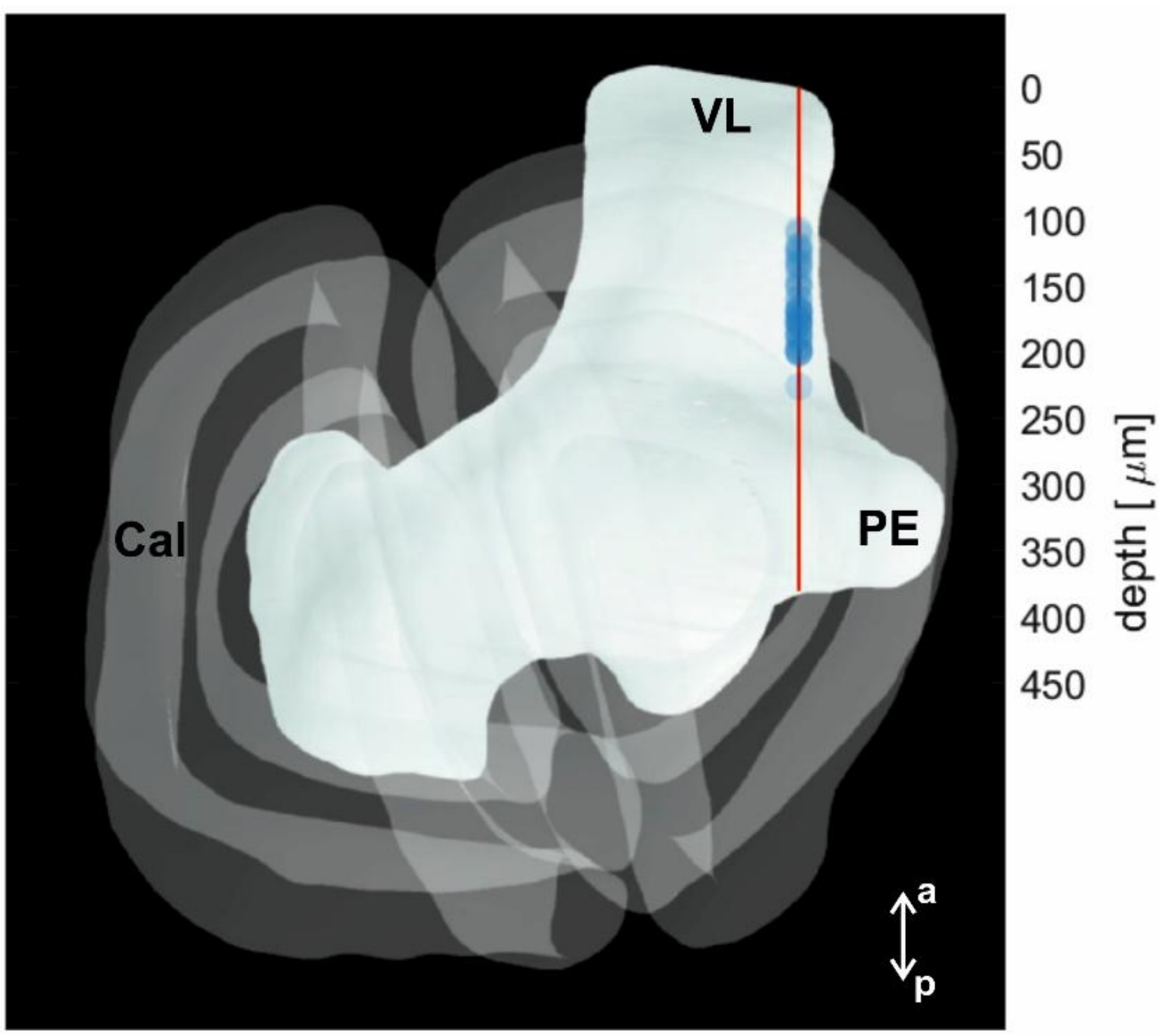

Figure S3: **Recording depth**: Visualisation of the mushroom body VL along the anteror-posterior (a-p) axis down to the peduncle (PE) (modified from Habenstein et.al 2023). The red line illustrates an idealized version of the approximate electrode insertion axis along the VL. Individual recording depth were illustrated by circles (blue). Note, that all recordings were restricted to the VL stopping before the PE (for individual depth cp. supplementary table S2).
